# The role of hybrid bone SPECT/CT imaging in the work-up of the limping patient: a symptom-based and joint-oriented review

**DOI:** 10.1186/s41824-018-0026-2

**Published:** 2018-04-23

**Authors:** H. K. Mohan, K. Strobel, W. van der Bruggen, G. Gnanasegaran, W. U. Kampen, T. Kuwert, T. Van den Wyngaert, F. Paycha

**Affiliations:** 1 Department of Nuclear Medicine, Sri Shankara Cancer Hospital and Research Centre, Bengaluru, India; 2 Department of Radiology and Nuclear Medicine, Lucerne Cantonal Hospital, Lucerne, Switzerland; 3 Department of Radiology and Nuclear Medicine, Slingeland Hospital, Doetinchem, The Netherlands; 4 Department of Nuclear Medicine, Royal Free London NHS Foundation Trust, London, UK; 5 Nuclear Medicine Spitalerhof, Hamburg, Germany; 6 Clinic of Nuclear Medicine, University Hospital Erlangen, Erlangen, Germany; 7 Department of Nuclear Medicine, Antwerp University Hospital, Wilrijkstraat 10, 2650 Edegem, Belgium; 8 Faculty of Medicine and Health Sciences, University of Antwerp, Wilrijk, Belgium; 9 Department of Nuclear Medicine, Hôpital Lariboisière, Assistance Publique-Hôpitaux de Paris, Paris, France

**Keywords:** Limping, Hip, Knee, Foot, Ankle, SPECT/CT, Bone scintigraphy

## Abstract

A vast spectrum of lower limb bone and joint disorders (hip, knee, ankle, foot) present with a common clinical presentation: limping. Too often this symptom generates an inefficient cascade of imaging studies.

This review attempts to optimise the diagnostic effectiveness of bone scintigraphy using the hybrid SPECT/CT technique in relation to the diagnostic clues provided by other imaging modalities, discusses the appropriate clinical indications, optimal scintigraphic procedures and illustrates updated image pattern-oriented reporting. Frequent lower limb bone and joint pathologies that can now be reliably diagnosed using hybrid bone SPECT/CT imaging will be reviewed.

Bone SPECT/CT can be an effective problem-solving tool in patients with persistent limping when careful history taking, clinical examination, and first-line imaging modalities fail to identify the underlying cause.

## Background

Limping is an abnormal pattern of locomotion caused by pain, weakness, or deformity and many conditions of the lower limb (hip, knee, ankle, foot) can present with this common clinical presentation. Too often this symptom generates a cascade of inefficient imaging studies, bone scintigraphy included, leading to unnecessarily high costs and delay in the final diagnosis. The introduction of hybrid SPECT/CT in nuclear medicine has drastically changed the diagnostic potential of bone scintigraphy in this setting. In this review, the clinical background of common hip, knee, and foot/ankle conditions will be summarized, the important technical considerations for performing bone SPECT/CT briefly reviewed, and potential applications of this technique illustrated with case material (Table 1).

**Table 1:** Overview of imaging features in potential applications of bone SPECT/CT in the limping patient

	CT features	SPECT features
Accessory ossicles	Presence of the accessory ossicle.	Symptomatic ossicles can show increased focal uptake (Chew 2010).
Ankylosing spondylitis	Erosions, sclerotic changes, subchondral bone changes, and bone formations at ligament insertions (Lacout et al. 2008).	Increased uptake at ligament insertions, vertebral bodies; facet and costotransverse/costovertebral joints (Fogelman et al. 2013).
Arthroplasty/osteosynthesis	Assessment varies according to type of arthroplasty or osteosynthesis. Features associated with pathology include: radiolucent zones (> 2 mm), periprosthetic fractures, endosteal scalloping, hardware breakage, component wear, heterotopic ossification, subluxation, dislocation, or soft-tissue masses or fluid collections (Roth et al. 2012).	Increased uptake at the bone-prosthesis interface suggests loosening, depending on implant positioning and fixation zones. Periprosthetic fractures or malunion show increased focal uptake at these sites. Diffuse uptake surrounding fixation screws is suspicious for loosening (Fogelman et al. 2013).
Avascular necrosis of hip	No abnormalities during early phase. Osteoporosis is the first visible sign, followed by clumping and distortion of the central trabeculae. An adjacent low-density region represents the reparative zone (Stoica et al. 2009). Best seen on plain radiographs, the crescent sign is a curvilinear subchondral radiolucent line corresponding with a subchrondral fracture.	Acute phase: photopenic area. Following restoration of blood flow: intense radiopharmaceutical uptake, indicating repair (Fogelman et al. 2013).
Benign bone tumors	Appearance varies according to histopathology. Important CT imaging characteristics include location within the bone, lesion margin, matrix proliferation, and periosteal reaction (Motamedi and Seeger 2011).	Tracer uptake can be highly variable between lesion types, ranging from poor uptake (e.g. solitary bone cyst, enchondroma), partial rim-shape uptake (e.g. aneurysmal bone cyst), to increased uptake (e.g. fibrous dysplasia, osteoblastoma). Variability within lesion types occurs (e.g. hemangioma) (Fogelman et al. 2013).
Plantar fasciitis	Bony spur may develop at the plantar aspect of the calcaneus and plantar fascia thickening can be visible (Chew 2010).	Focal calcaneal hyperemia on blood pool images, extending into the proximal plantar fascia in more severe disease. Delayed images show focal calcaneal uptake (Frater et al. 2006).
Femoral acetabular impingement (FAI)	Cam type: non-spherical femoral head or lack of neck concavity. Pincer type: deep or overhanging acetabulum. Labral ossification, joint space narrowing, and osseous hypertrophy of the acetabular rim develop in chronic FAI (Morrison and Sanders 2008).	Hyperemia on blood pool images. Delayed images show a “reverse C” pattern of joint uptake. Intense focal uptake in the lateral and inferomedial aspect of the femoral head indicate impingement (Fogelman et al. 2013).
Heterotopic ossification (HO)	Muscle swelling containing calcification: amorphous (poorly defined with no trabecular structure), immature (initial trabecular formation with poorly defined margins), or mature (well-defined cancellous bone with cortical outline) (Zagarella et al. 2013).	Flow and blood pool is positive 2–3 weeks after injury. Delayed images become positive about 1 week later. Peak activity occurs a few months after injury, followed by progressively decreasing uptake with normalization at 6 to 12 months after injury (Shehab et al. 2002).
Insufficiency/stress fracture	Linear sclerosis possibly with subtle focal cortical interruption or step-off (Morrison and Sanders 2008).	Typical appearance is of hyperemia with intense uptake on late images at the site of injury (Fogelman et al. 2013).
Osteoarthritis	Uneven loss of articular space, subchondral sclerosis, osteophytes, and subchondral cysts. Absence of osteoporosis, ankylosis, and erosions (Chew 2010).	Usually no hyperemia. Late images typically show a combination of diffuse articular uptake depending on disease severity, and (multi)focal uptake at the joint edges or weight-bearing surfaces (Boegard et al. 1999; Fogelman et al. 2014).
Osteochondritis dissecans	Subchondral irregular areas of increased and decreased density, with normal, thinned, or eroded overlying articular cartilage (Bloem and Sartoris 1992).	Uptake depends on the age of the lesion. Acute lesions can show subtle focal hyperemia, with more intense delayed uptake at the site of injury (Fogelman et al. 2014).
Osteoid osteoma	Osteolucent focus at the nidus, with or without a dense central mineralized focus. Surrounding extensive fusiform sclerosis is typical in long bones (Motamedi and Seeger 2011).	Typical findings show a vascular lesion on blood pool images and intense focal uptake on the delayed image (Fogelman et al. 2014).
Spontaneous osteonecrosis of the knee	No changes in early disease. Later, subtle flattening of the condyle develops, followed by appearance of a radiolucent area (crescent or rim sign) indicating segmental necrosis of subchondral bone.	Intense uptake on blood pool and late phase images early after onset for up to 6 months. This is followed by a gradual decrease of blood pool uptake, but persisting positive late phase images for up to 2 years (Elgazzar 2004).
Tarsal coalition	Abnormal osseous continuity of two bones, or more subtle abnormalities in non-osseous coalitions (joint space narrowing, minimal marginal reactive osseous changes). Subchondral joint cysts can occur in an otherwise non-arthritic appearing foot (Lawrence et al. 2014).	Focal uptake at the site of coalition and at any sites complicated by osteoarthritis (Fogelman et al. 2013).
Tendinopathies	General or localized swelling of the tendon may be observed on CT. When partial rupture has occurred focal intratendinous inhomogeneities with lower attenuation compared to the surrounding tissue become apparent (Kalebo et al. 1990).	Increased uptake on the flow and blood pool images (Pelletier-Galarneau et al. 2015).
Tophaceous gout	Peri-articular rat-bite erosions with overhanging edges. Non-calcified dense soft-tissue tophi. Tendon thickening may occur (Morrison and Sanders 2008).	Blood pool and delayed images typically show intense increased uptake in the affected joints (Fogelman et al. 2014).
Transient osteoporosis	Diffuse osteopenia of the affected area without crescent sign or collapse of the femoral head.	Blood pool and delayed images typically show increased uptake with varying intensity in the femoral head extending into the femoral neck and intertrochanteric region, without focal cold spots (Gemmel et al. 2012).

## Hip

### Clinical context

The hip joint is a major weight-bearing ball-and-socket type of joint that is subjected to significant stresses during activities of daily living. Therefore, it is not surprising that more than 40% of adults over age 65 experience hip pain at some point, and also 5–10% of athletes report an episode of hip pain on a yearly basis. These numbers illustrate that hip pain is indeed a very common problem.

**Fig. 1:**
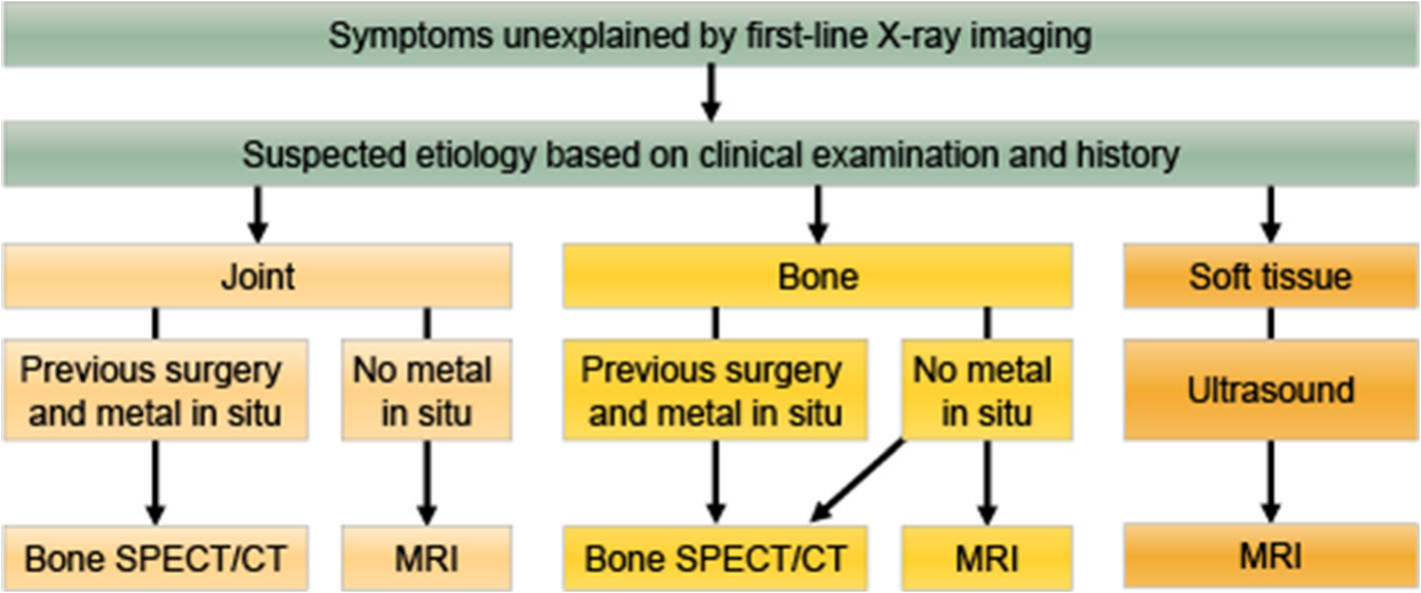
Imaging algorithm chronic hip pain. Proposed imaging algorithm for chronic hip pain unexplained by clinical examination and history taking

A thorough history taking and clinical examination are of crucial importance when assessing a patient referred for hip pain, including assessing the presence of “red flags” symptoms, as will be discussed later. The clinical examination of the adult (athletic) hip combined with the disease history will provide the clinician with a working diagnosis in approximately 80% of patients (Braly et al. 2006). In the 10–20% of patients in whom a working diagnosis cannot be made using this initial assessment, additional diagnostic procedures will be required. Algorithmic approaches have been published that can be applied in a primary care setting and these propose magnetic resonance imaging (MRI) if plain X-rays and conservative therapy are ineffective (Margo et al. 2003). Similarly, the American College of Radiology Appropriateness Criteria are heavily reliant on MR imaging, even in the setting of co-existent chronic low-back, pelvic, hip, or knee pathology (Mintz et al. 2017). Nevertheless, the advent of hybrid imaging in nuclear medicine has transformed bone scintigraphy into a comprehensive tool that not only enables whole-body skeletal assessment, in contrast to MRI, but also allows for a detailed regional evaluation using targeted SPECT/CT acquisitions. The major driver for this improvement in impact on patient management has been the increase in specificity, a traditional weak-point of conventional bone scintigraphy (Dobrindt et al. 2015). Current indications for the use of bone SPECT/CT in the painful adult hip include, assessment of joint replacements for loosening/infection, heterotopic ossification, impingement, stress fractures, avascular necrosis, hip pain unexplained by other imaging modalities, and ruling-out metastatic disease or systemic arthropathy. Of course, any imaging strategy should be guided by the findings on clinical examination and symptom history (Fig. 1).

### Technical considerations

In concordance with the EANM guideline for bone scintigraphy, static planar blood images are routinely recommended (256 × 256 matrix; LEHR or LEGP collimators; 2–5 min per view), followed by planar delayed images of the hip area (or as part of a whole-body acquisition) (256 × 256 matrix; LEHR collimators; 5 min/500 kcts per view). Typical SPECT/CT acquisition parameters for SPECT (128 × 128 matrix; 128 angles; 20 s/angle), combined with localization CT (2.5–40 mA; 80–130 keV; 1–5 mm slice thickness), or diagnostic CT (40–335 mA; 80–130 keV; 0.33–2.0 mm slice thickness) have been proposed, but may vary and should be optimized according to the type of hybrid device (e.g. flat panel CT detector, available dose reduction techniques, etc) (Van den Wyngaert et al. 2016). If available, the use of metal artefact reduction technology is strongly recommended for all prostheses imaging (Abdoli et al. 2016).

### Applications of SPECT/CT

#### Arthroplasty

Complications, such as loosening or infection, are common problems after hip arthroplasty and bone SPECT/CT is increasingly being used as the second step imaging modality if conventional X-rays are equivocal (Strobel et al. 2012). Blood pool imaging remains a valuable technique to assist in differentiating loosening from infection. The absence of increased blood pool uptake would argue against the presence of overt infection, even though low-grade processes cannot be entirely excluded (Collier et al. 1996). In addition, the CT part of the study may also provide valuable clues suggesting an infectious cause of loosening, for example adjacent soft-tissue extension, fluid collections, or fistula tracts. Similarly, increased uptake at the bone-prosthesis interface – in contrast to uptake in the surrounding bone – in areas associated with prosthesis fixation requires careful assessment of the appearance on CT to exclude loosening (Tam et al. 2014). Using this approach, bone SPECT/CT can identify the source of pain and impact management in approximately 65% of patients with pain after hip arthroplasty and negative conventional imaging (Dobrindt et al. 2015; Berber et al. 2015). Nevertheless, dedicated follow-up infection imaging may be required, but is beyond the scope of this review.

#### Femoral acetabular impingement

**Fig. 2:**
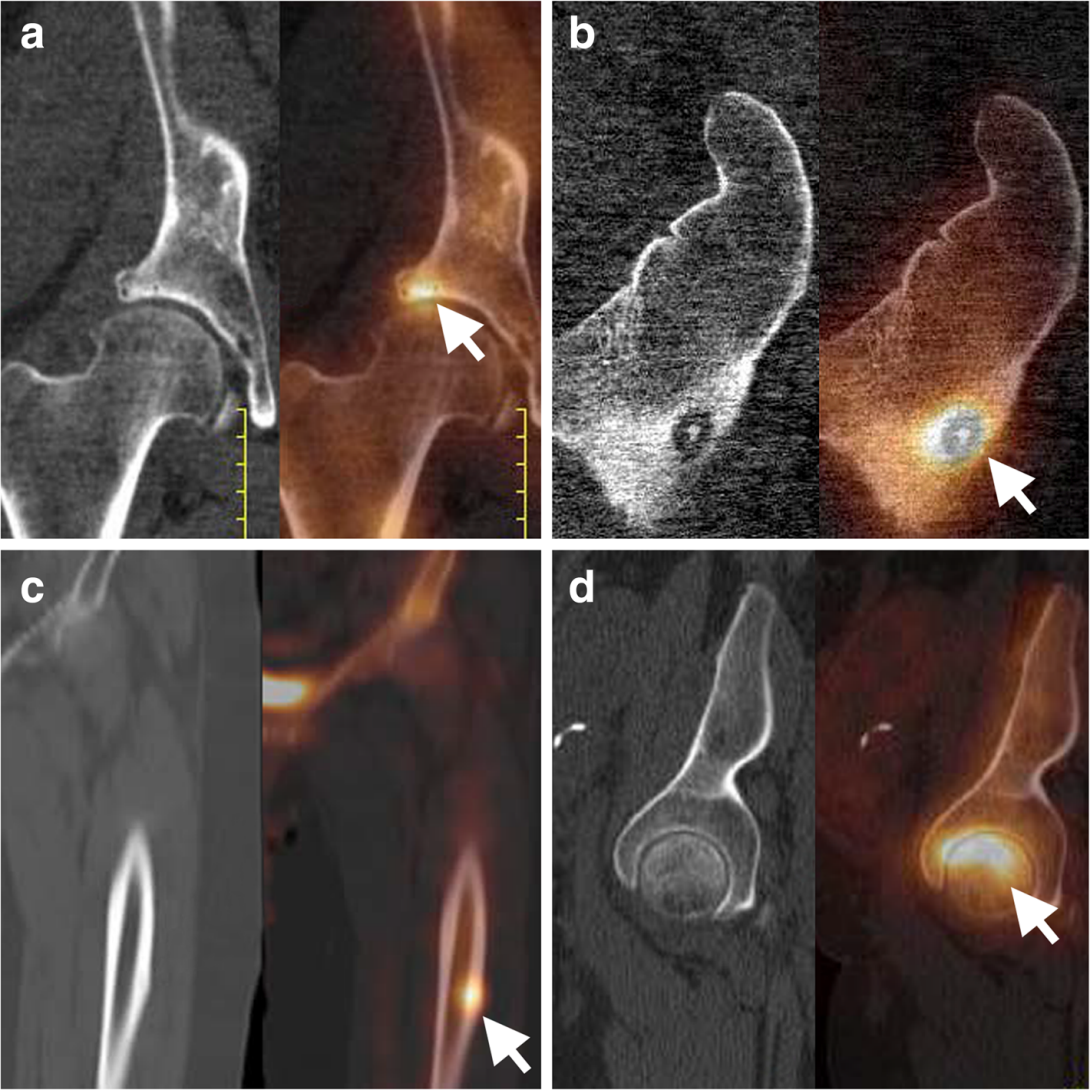
Bone SPECT/CT imaging of the painful hip. Illustration of bone SPECT/CT applications in imaging the painful hip. **a** femoro-acetabular impingement of the right hip, **b** subtrochanteric osteoid osteoma of the left femur, **c** chronic bisphosphonate use induced stress fracture of the left femur, and **d** avascular necrosis of the femoral head (reparative phase)

Not only the normal degenerative process, but also an anatomical structural abnormality of the acetabular cup or the femoral head can cause accelerated wear. While MR arthrography remains the preferred technique to diagnose impingement, as it also allows assessment of the labrum and cartilage, bone SPECT/CT can be an alternative when MR imaging is not possible. The finding of increased uptake in the superior femoral neck associated with osteoarthritic uptake involving the superior hip joint suggests the diagnosis of either cam or mixed cam-pincer impingement (Fig. 2a). The combination of SPECT/CT enables assessment of the femoral and acetabular configuration in order to identify cam and pincer type abnormalities (Lee et al. 2008).

#### Osteoid osteoma

Osteoid osteoma is a painful benign bone lesion typically occurring in young adults and characterized by increased focal uptake on late-phase bone scan, with a sclerotic nidus on CT and increased vascularity on blood pool images. It can be accurately characterized using bone SPECT/CT (Fig. 2b), whereas with MR imaging osteoid osteoma can be easily misdiagnosed, especially in complex anatomic regions like spine and foot, because of confusing surrounding edema and more difficult assessment of the typical “nidus” compared with (SPECT)/CT.

#### Stress fractures

The chronic use of bisphosphonates, in particular for the treatment and prevention of osteoporosis, has been associated with low-energy atypical subtrochanteric and proximal diaphyseal femoral fractures (Patel et al. 2016). These may be preceded by chronic stress fractures which can be subtle on anatomic imaging, whereas they can be easily identified using bone SPECT/CT (Fig. 2c).

#### Avascular necrosis of hip

In patients with contraindications for MRI and metallic implants, bone SPECT/CT has superior diagnostic properties for diagnosing avascular necrosis of the hip when compared with SPECT-only imaging (Fig. 2d) (Luk et al. 2010).

#### Heterotopic ossification

While it is usually advised to wait at least 1 year after arthroplasty before performing bone scintigraphy, an exception can be made when heterotopic ossification is suspected, as this condition affects the adjacent soft tissues where no physiological tracer uptake is expected (Shawgi 2012). Bone SPECT/CT can be of additional value to demonstrate pseudarthrosis of the heterotopic ossification site with adjacent bone. Moreover, bone scintigraphy helps in assessing the extent and activity of the disease, which is important for planning and timing surgical treatment (Shehab et al. 2002).

#### Transient osteoporosis

Transient osteoporosis of the hip is an idiopathic, self-limiting syndrome characterized by progressive or sudden onset pain in the groin, slightly reduced mobility of the hip, and localized radiographic osteopenia. The condition usually shows spontaneous recovery within 2 to 9 months (Gemmel et al. 2012). Plain radiographs show osteopenia in about 70% of cases, without any signs of erosion, joint space narrowing, or subchondral collapse. The typical patterns of bone SPECT and CT are summarized in Table 1. MRI classically demonstrates a pattern of diffuse bone marrow edema varying in subtlety, giving rise to the alternate name of this condition: transient bone marrow edema syndrome.

## Knee

### Clinical context

The knee is a synovial modified hinge joint that consists of three articulations: the tibiofemoral joint, the tibiofibular joint and the patellofemoral joint. Today, MR imaging is a very powerful tool for the assessment of the painful knee joint, in particular it provides exquisite detail of the cruciate ligaments, the collateral ligaments, menisci, synovia and cartilage. MR imaging in osteoarthritis may typically show bone edema, loss of cartilage, degenerative tears in the menisci, Baker’s cysts, and effusion in the knee joint. MRI is also regularly performed for the local assessment of malignant bone tumors like osteosarcoma, and is a useful technique to evaluate the extension of osteomyelitis. However, MR imaging also has contraindications, including severe claustrophobia, some pacemakers, metallic foreign bodies, and renal insufficiency precluding the administration of gadolinium contrast. Also, MR images can be severely degraded by susceptibility artefacts when metallic implants are present, making it difficult to assess the bone-prosthesis interface, even despite the introduction of metal artefact reduced MR sequences (Hayter et al. 2011). While whole-body imaging is possible with MRI, this is only rarely implemented in daily routine practice in most centers. Finally, while MRI is very good for bone marrow assessment, it is limited in the assessment of cortical bone.

**Fig. 3:**
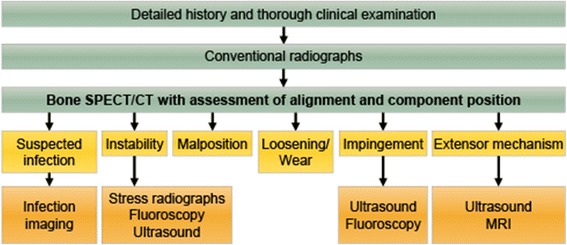
Imaging algorithm painful knee arthroplasty. Imaging algorithm for assessing the painful knee arthroplasty using bone SPECT/CT as second-line modality after conventional radiographs. Depending on the differential diagnosis generated by SPECT/CT selected follow-up imaging techniques may be required. Modified from Hirschmann et al. (Hirschmann et al. 2013)

**Fig. 4:**
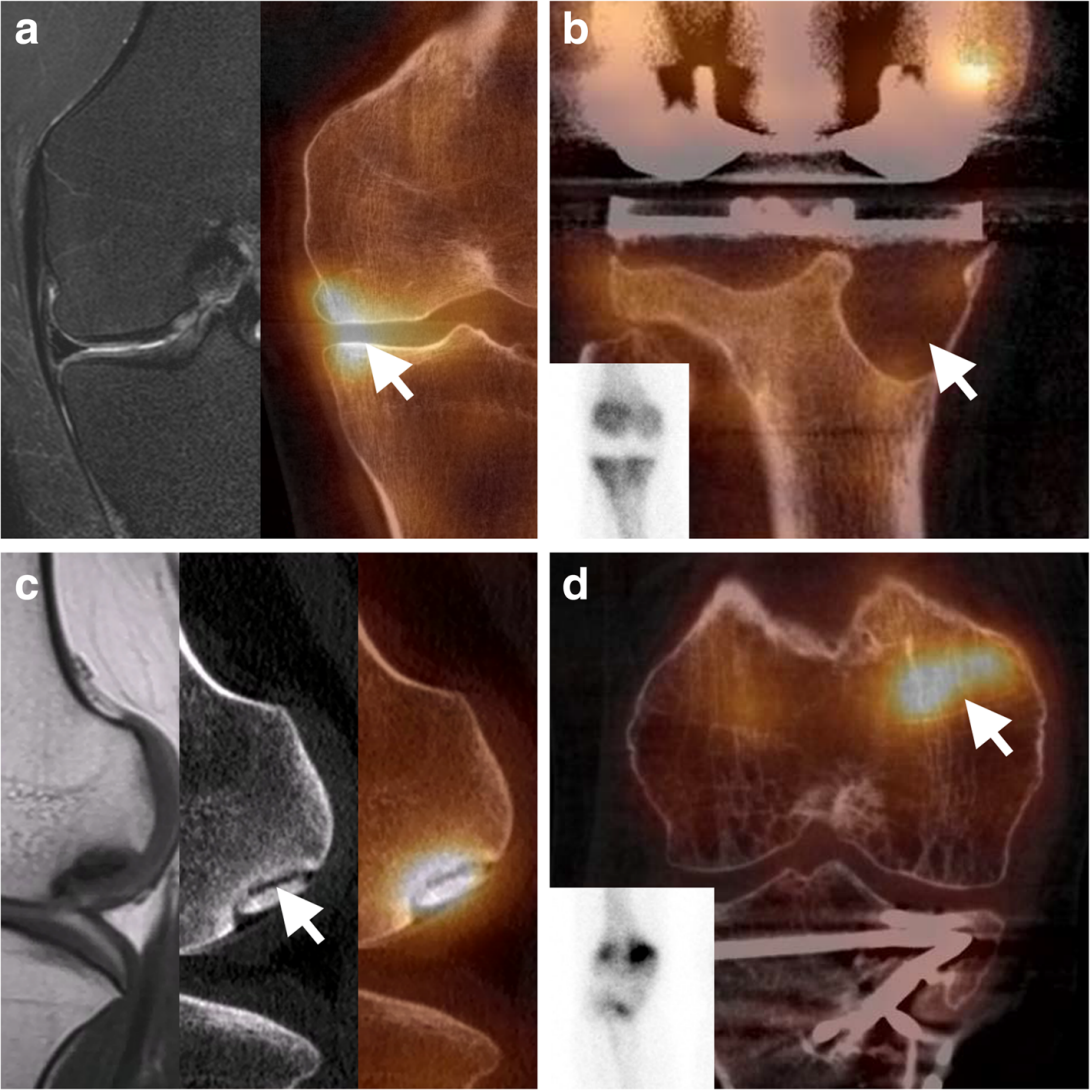
Bone SPECT/CT imaging of the painful knee. Illustration of bone SPECT/CT applications in imaging the painful knee. **a** medial compartment overloading demonstrated on bone SPECT/CT but not visible on MR STIR imaging, **b** loosening of arthroplasty due to large granulomas causing only diffuse non-specific uptake on planar imaging (inset), **c** example of osteochondritis dissecans with a gap surrounding the fragment not visible on MRI, **d** complex osteosynthesis of the proximal tibia after trauma with pain recurrence due to stress fracture of the distal femur on planar (inset) and SPECT/CT, but unsuspected on anatomical imaging

The strengths of bone scintigraphy and SPECT/CT are the combination of morphologic and metabolic information. Moreover, a whole-body study can easily be combined with a dedicated knee imaging SPECT/CT protocol, the technique can be performed in the presence of metallic implants, and a detailed assessment of the cortical bone structures is possible. Recently, bone SPECT/CT has been proposed as second-line imaging technique to evaluate painful knee arthroplasties (Fig. 3). The uptake on bone scintigraphy and the presence of bone marrow edema on MRI contribute independent diagnostic information and represent distinct biological processes (Strobel et al. 2014). Therefore, one is not interchangeable by the other, as illustrated by the study of Buck et al. showing that uptake on scintigraphy is more sensitive compared to bone marrow edema on MRI to detect mechanical bone overload in patients with chronic medial knee pain (Fig. 4a) (Buck et al. 2009).

There are many indications left for radionuclide imaging of the knee in the era of MRI, with major indications comprising the patient with inconclusive conventional imaging or inability to undergo MRI, after arthroplasty, infection, and bone tumors. To achieve these benefits, it is crucially important to leverage the full diagnostic power of CT as part of the SPECT/CT study.

### Technical considerations

With respect to bone SPECT/CT studies, high resolution CT images of the knee should be acquired in order to fully compete with MRI. To that end, a value in the lower range of slice thickness settings proposed by the EANM bone scintigraphy guideline should be used (e.g. 0.33 mm) and settings with higher range values for mAs and kV may be needed (Van den Wyngaert et al. 2016). However, this does not necessarily mean that a high dose of radiation has to be delivered to the patient, as special detector design (e.g. flat panel CT) and dose reduction techniques (e.g. iterative reconstruction algorithms) are nowadays available. Using these advances, the additive radiation exposure can be in the range of only 10% on top of the dose received by bone scintigraphy.

### Applications of SPECT/CT

#### Arthroplasty

It is well known that there can be persistent residual tracer uptake on bone scintigraphy many years after knee arthroplasty, in particular surrounding the tibial component, even in asymptomatic patients. However, the introduction of SPECT/CT has led to a major improvement in the specificity of the technique and it has become a very valuable tool in the assessment of the painful knee arthroplasty (Hirschmann et al. 2013). For example, CT can readily identify a granuloma as the cause of otherwise non-specific diffuse uptake on bone scintigraphy (Fig. 4b). Also, focal uptake below the medial part of the tibial component without any abnormalities on CT can be dismissed as physiological with a higher degree of confidence. As shown in Fig. 3, bone SPECT/CT should not be the first line imaging modality, but only after conventional radiographs, detailed history and thorough clinical examination fail to produce a diagnosis (Hirschmann et al. 2013). Other diagnostic follow-up examinations and procedures may be required after bone SPECT/CT, for example to rule out infection or assess ligaments and soft tissue structures. Using this approach, bone SPECT/CT has proven to be a reliable technique to detect loosening (Abele et al. 2015) or progression of patellofemoral osteoarthritis (OA) (Hirschmann et al. 2015), significantly impacting management in 85% of patients. Infection imaging using anti-granulocyte antibodies has also benefited from the introduction of SPECT/CT, increasing overall accuracy compared to planar imaging or SPECT only imaging (Graute et al. 2010).

A novel application is to use bone SPECT/CT combined with arthrography, especially in patients who have metal material present precluding good MR imaging. When injecting iodine containing contrast material into the knee joint, a detailed assessment of the integrity of cartilage, the detection of loose bodies and a rough evaluation of meniscus and ligaments is possible (Huellner and Strobel 2014).

#### Osteochondritis dissecans

For the assessment of osteochondritis dissecans the correlation of a hotspot on SPECT with the anatomical information on CT is essential. While it can be used to assess the cartilage with good detail, CT can also detect any gaps between the loose fragment and adjacent bone (Fig. 4c).

#### Osteosynthesis

After osteotomy, bone SPECT/CT can be used to assess the activity in the osteotomy and assess whether the bone fragments have properly integrated. Also, X-ray can be difficult to interpret after osteosynthesis of traumatic lesions, whereas SPECT/CT can guide image interpretation towards areas of pathologically increased bone turnover (Fig. 4d) (Huellner and Strobel 2014).

#### Benign bone tumors

While FDG-PET/CT and PET/MRI are probably of more utility in the assessment of malignant bone tumors like osteosarcoma, bone SPECT/CT can be useful to characterize focal areas of increased uptake on bone scintigraphy as definitively benign lesions. For example, the typical appearance of a non-ossifying fibroma on CT will obviate further investigations for what otherwise would have been an equivocal bone scan report. In addition, it can complement MRI when for example the integrity of cortical bone adjacent to an aneurysmal bone cyst cannot be adequately assessed.

#### Spontaneous osteonecrosis of the knee

Spontaneous osteonecrosis of the knee (SONK) predominantly affects women above 60 years of age and most often occurs unilaterally in the medial femoral condyle, even though any part of the knee can be involved. Symptoms include abrupt onset pain, localized tenderness, stiffness, effusion, and restricted motion (Elgazzar 2004). These features distinguishes SONK from secondary forms of osteonecrosis, which typically affect younger patients, and have a more insidious onset with less severe symptoms, but are often bilateral. The exact pathogenesis remains unclear, but subchondral insufficiency fractures in osteopenic bone may initiate a cascade of bone marrow edema leading to focal ischemia. While initial stages of the condition are considered to be reversible, with no radiographic abnormalities (stage 1) or only slight flattening of the condyle (stage 2), higher stages are associated with irreversible cartilage and subchondral bone destruction, leading to articular collapse (stage 4). Bone scan typically shows intense uptake on blood pool and late phase images up to 6 months after onset, followed by a gradual decrease of blood pool uptake, but persisting positive late phase images for up to 2 years. Three to 6 months after onset, a radiolucent lesion appears on plain radiographs and CT (crescent or rim-sign) indicating segmental necrosis of subchondral bone (stage 3). Bone SPECT/CT is most useful in guiding the diagnosis in the early stages of SONK because of the very subtle changes visible on anatomic imaging. MR imaging of stage 1 is considered non-specific, but from stage 2 changes on MRI are usually typical for osteonecrosis.

#### Synovitis and inflammatory joint disease

Synovitis due to many different inflammatory conditions (including rheumatoid arthritis) or even in late stage osteoarthritis commonly affects the knee joint. With increasing severity of the condition, radiographic findings include joint effusion, joint space narrowing, subchondral osteolysis or erosions, cyst formation, and ankylosis. However, the role of CT in the assessment of rheumatoid arthritis and synovitis is limited as ultrasound and MRI are preferred (Sommer et al. 2005). Blood pool images are an indispensable tool for the assessment of synovitis on bone scintigraphy and typically show increased synovial uptake, even in very early stages where radiographic findings may still be absent. The delayed phase tends to reflect subchondral and entheseal uptake, extending along the synovial reflections of the joint in severe cases (Fogelman et al. 2013). Together, this diffusely increased tracer uptake in the synoviosubchondral bones of the whole knee joint and the patella, is referred to as the “wrapped bone” appearance (Bahk 2017). Inflammatory joint disease should be part of the differential diagnosis in a limping patient and SPECT/CT can assist in narrowing the potential causes of pain, including inflammation at an early stage.

## Foot

### Clinical context

**Fig. 5:**
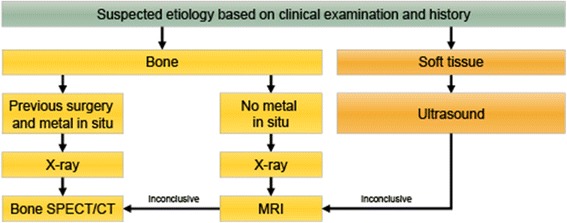
Imaging algorithm painful foot and ankle. Imaging algorithm for assessing the painful foot and ankle. Modified from Mohan et al. (Mohan et al. 2010)

Foot pathology is responsible for 10–20% of patients presenting with limping in routine clinical practice. Foot bone and joint pain may be related to mechanical conditions caused by weight bearing or as a first sign of non-traumatic systemic disease (e.g. rheumatoid arthritis). In addition, the foot is a complex anatomic region, merging form and function with many intricate relationships. Next to conventional radiographs, MR imaging is preferred for the assessment of foot and ankle because of the excellent visualization of bone and soft-tissues. Historically, the contribution of bone scintigraphy to diagnosing foot and ankle disease has been restricted by the limited resolution and low specificity. However, hybrid bone SPECT/CT has also significantly improved the value of radionuclide bone imaging of this anatomical region, proving its superiority over planar imaging (Upadhyay et al. 2017). Current applications of bone SPECT/CT in foot and ankle pathology typically include patients with chronic pain symptoms that are insufficiently explained by clinical findings and conventional imaging. These include locating active sites of osteoarthritis, coalitions, osteoid osteoma, occult stress fractures, tendinitis, plantar fasciitis, and impingement syndromes (Pelletier-Galarneau et al. 2015; Farid et al. 2010; Williams et al. 2012). Therapy response assessment after surgical interventions like assessment of successful arthrodesis are further promising applications. Recent data suggest that bone SPECT/CT has MRI-comparable diagnostic performance for symptomatic lesions in ankle and foot pain patients and depending on the clinical context, the technique can be proposed as second or third-line imaging modality (Fig. 5) (Ha et al. 2015).

### Technical considerations

As for any radionuclide study, good clinical information and availability of the findings of other imaging techniques are a prerequisite for bone SPECT/CT. From a technical viewpoint, optimal patient positioning and immobilization of the feet respecting the anatomical position are paramount during image acquisition. In order to avoid misregistration artefacts, the patient should be placed in a painless position using radiotransparent and comfortable supports. As for imaging of the knee, a low value for CT slice thickness (e.g. 1.25 mm) is preferred (Van den Wyngaert et al. 2016). Given the complex regional anatomy, blood pool images should also be acquired using the SPECT technique (e.g. 8 s per step; 72 steps over 360°), combined with ultralow-dose CT (e.g. 80 kV and 20 mAs). This can be of particular use to detect ligamentous injuries that typically present with increased uptake on blood pool SPECT images whereas late phase images will not show bone involvement. An additional CT acquisition is required as it is currently not possible to reliably merge blood pool SPECT series with late phase images.

Contemporary SPECT/CT viewing solutions allow for oblique orientation according to the axis of interest, which is mandatory in order to perform a comprehensive assessment of the imaged limb. In the same respect, volume rendering techniques can be very useful in order to pinpoint the abnormality relative to the entire foot anatomy.

### Applications of SPECT/CT

#### Osteoarthritis

**Fig. 6:**
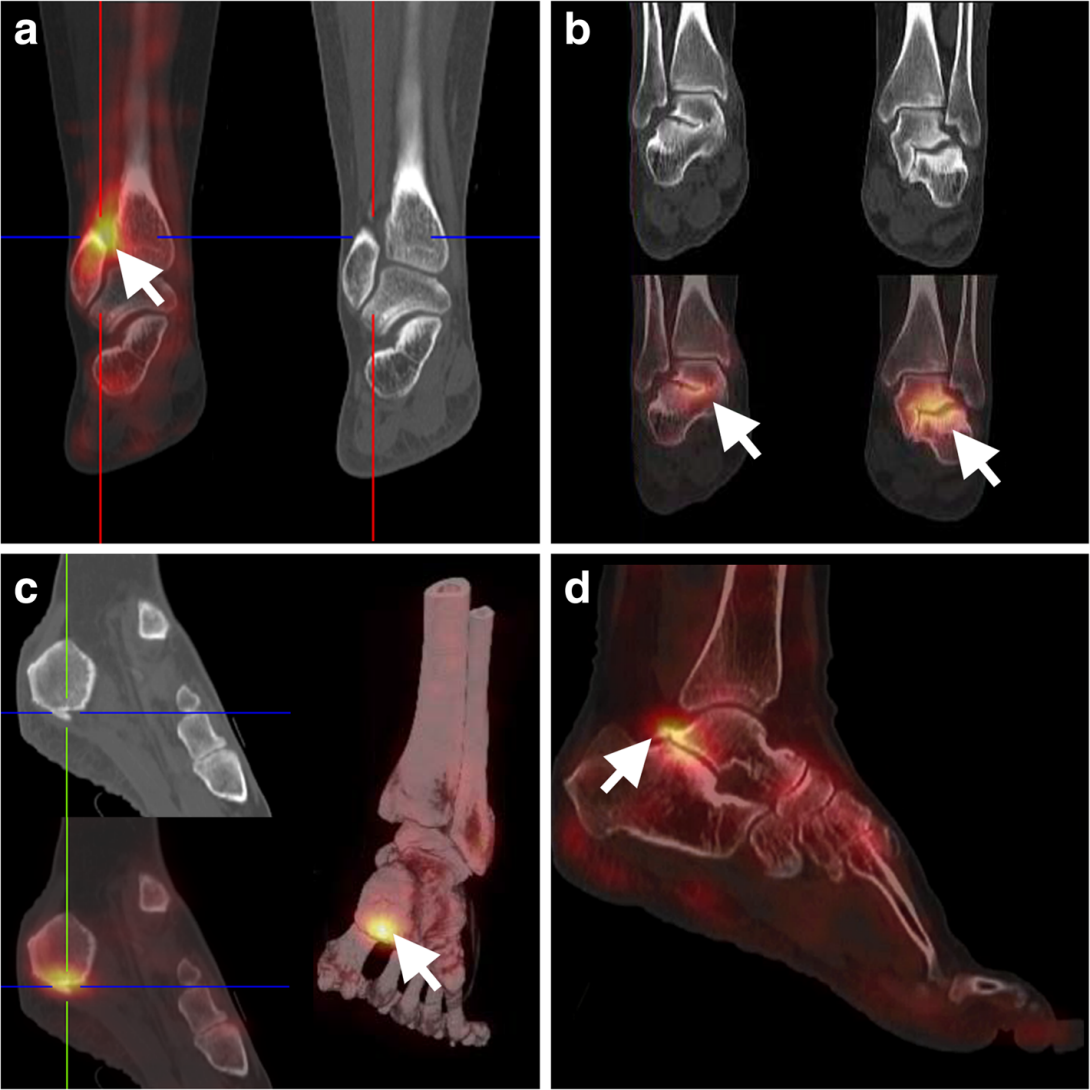
Bone SPECT/CT imaging of the painful foot/ankle. Illustration of bone SPECT/CT applications in imaging the painful foot and ankle. **a** degenerative changes in the distal tibiofibular syndesmosis complex after trauma, **b** partial ankylosis of the right subtalar joint and syndesmosis with subchondral sclerosis of the left subtalar joint in a patient with ankylosing spondylitis, **c** fasciitis plantaris, **d** os trigonum syndrome

Osteoarthritis of the foot and ankle are common conditions and the clinical significance of tarsal tracer uptake may have been underestimated in the era of planar bone scintigraphy. In particular mid-foot problems appear good targets for bone SPECT/CT imaging (Pagenstert et al. 2009). Also, evaluation of degenerative changes in the distal tibiofibular syndesmosis complex after trauma has improved with the introduction of bone SPECT/CT (Fig. 6a). In addition, the technique can be of use to guide the optimal site for intra-articular infiltrations (Parthipun et al. 2015). Finally, literature data supports the use of bone SPECT/CT when pain recurs after arthroplasty or arthrodesis for degenerative joint disease (Biersack et al. 2012; Mason et al. 2015).

#### Osteochondral lesions

In osteochondral lesions, SPECT/CT can contribute valuable information in addition to MRI on the extent of the lesion, on determining the most active lesion in case of multifocal disease, and on selecting patients for surgical intervention (Meftah et al. 2011).

#### Insufficiency fracture

The typical bone SPECT/CT pattern of insufficiency fractures consists of increased focal or linear uptake on SPECT, no apparent changes or discrete fracture lines on CT, and MRI findings ranging from moderate periosteal edema on STIR images, to marrow changes on T2 and later T1 images, up to a visible fracture line.

#### Post-traumatic injuries

Persisting pain after ankle inversion injuries is common and causes include undetected syndesmotic sprain, talar osteochondral lesions, and stress fractures, all of which can be diagnosed using bone SPECT/CT (Pelletier-Galarneau et al. 2015). Syndesmotic sprain presents as increased tracer uptake in the posterior tibial region and interosseous membrane on delayed phase images, while changes on plain radiographs and CT can be subtle. Similarly, talar osteochondral lesion demonstrate increased subarticular uptake that can also involve the entire posterior half of the talus.

#### Tarsal coalition

Tarsal coalitions are congenital anomalies consisting of complete or partial unions between two or more bones of the midfoot or hindfoot. Patients typically present with foot pain in adolescence. The vast majority of coalitions occur in the calcaneonavicular (45%) or talocalcaneal (45%) joint. SPECT/CT is very helpful in the assessment of the fusion type (bony, cartilaginous or fibrous coalition) and the grade of scintigraphic activity.

#### Ankylosing spondylitis

Ankle pain may be the harbinger of systemic inflammatory diseases such as ankylosing spondylitis. Focally increased uptake in the talocalcaneal joints correlating on CT with partial ankylosis with or without subchondral sclerosis can provide important clues for a broader systemic disease etiology (Fig. 6b).

#### Plantar fasciitis

The typical scintigraphic pattern consists of increased uptake in the medial calcaneal tubercle correlating with a spur on CT (Fig. 6c). While it often presents as an isolated condition, it can also be associated with ankylosing spondylitis.

#### Tendinopathies

Posterior tibial tendon dysfunction (PTTD) is characterized by pain and swelling over the course of the tendon at the level of the medial malleolus and navicular insertion (Pelletier-Galarneau et al. 2015). It can be an overuse injury in runners caused by repetitive trauma inducing an inflammatory response. Flow and blood pool images show increased uptake along the tendon and medial malleolus, extending to the navicular (Kannangara et al. 1999). Another frequent cause of posterior foot pain is Achilles tendon injury.

#### Accessory ossicles

There are many possible accessory ossicles of the feet and while most often asymptomatic, they can be associated with pain and increased uptake on bone SPECT/CT (e.g. os trigonum syndrome; Fig. 6d).

#### Tophaceous gout

Caused by urate deposition, tophi are soft tissue nodules which appear in periarticular soft tissues and occur together with bone erosions with sclerotic margins producing typical punched-out lesions with overhanging cortical edges. Unlike other arthropathies such as rheumatoid arthritis, joint space and bone mineral density are well preserved until late in the disease course. Of note, differential diagnosis with tumoral calcinosis is possible because urate crystals do not exhibit tracer uptake in contrast to calcium deposits.

## Referred pain

### Epidemiology

In contrast to referred visceral pain, there is a lack in standardization of the definition of referred pain in limping patients, with literature reports often mixing referred pain with other types of pain. It is therefore important to note what is not considered referred pain in this context: radicular pain (cause by nerve irritation by for example spinal disc herniation and producing sciatica symptoms in well localized dermatomes), poorly localized pain (describes the inability to pinpoint the location of pain), and complex regional pain syndrome (CRPS) (generally poorly understood disease mechanism that presents with different symptoms and requires other treatments than referred pain).

The mechanism of referred pain is poorly understood but most experts agree that referred pain can be described as pain perceived in a different or wider area than the site of origin. Nociceptive, neurogenic or psychological factors are likely to contribute and the pain is usually felt as a well localized deep pain. Central pain perception mechanisms can be involved, including sensitization and cortical and subcortical reorganization (Latremoliere and Woolf 2009; Roussel et al. 2013). These processes cause pain perception to become disconnected from the actual pain stimulus, and in case of the limping patient this mostly involves the adjacent joint. A summary of possible sources of referred pain is given in Table 2.

**Table 2:** Summary of causes of referred pain

Area of pain sensation	Referred pain etiology
Knee	Low back • Facet joint arthropathy • Discopathy • Malignancy Hip • Osteoarthritis • Slipped capital femoral epiphysis • Trauma Sacroiliac joint • Osteoarthritis • Malignancy
Thigh	Low back • Facet joint arthropathy • Discopathy • Malignancy Knee • Osteoarthritis Sacroiliac joint • Osteoarthritis • Malignancy Soft tissue • Kidney • Ureter
Low back	Knee • Osteoarthritis (infrequent cause of referred pain to the low back) Sacroiliac joint • Osteoarthritis • Malignancy Soft tissue • Kidney • Ureter • Liver • Pancreas • Bowel • Aortic aneurysm

### Technical considerations

Late-phase planar whole-body images remain a recommended acquisition and important asset when performing bone scintigraphy, especially in the context of referred pain. In any case, at least the joint adjacent to the symptomatic area should be imaged in order to screen for abnormalities (Van den Wyngaert et al. 2016).

### Applications of SPECT/CT

#### Lumber spine facet joint arthropathy

**Fig. 7:**
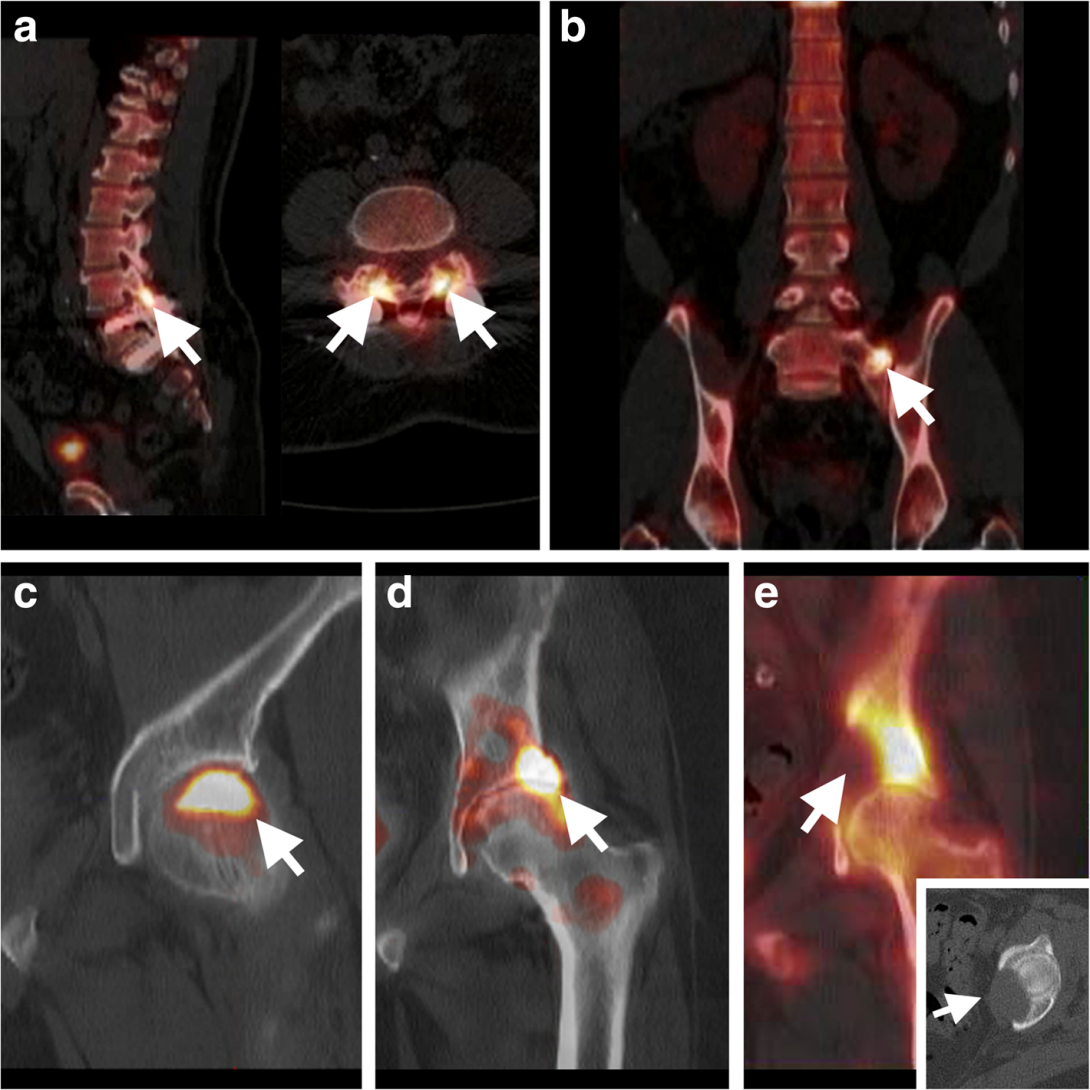
Bone SPECT/CT imaging of referred pain. Illustration of bone SPECT/CT applications in imaging of conditions associated with referred pain: **a** degenerative facet joint arthropathy in the segment above spinal fusion, **b** symptomatic Bertolotti’s syndrome, and focal uptake in the hip where SPECT/CT imaging allows differentiation of **c** avascular necrosis, **d** osteoarthritis, and **e** an osteolytic bone metastasis and soft-tissue component (inset)

For referred pain originating from degenerative facet joint disease in the low back (Fig. 7a), bone SPECT/CT has been shown to contribute to identifying the pain generator, can be used to guide facet joint injections and predict patient outcome after treatment (Pneumaticos et al. 2006; Al-Riyami et al. 2017).

#### Bertolotti’s syndrome

Bertolotti’s syndrome is low back pain associated with a transitional lumbar vertebra and asymmetric fusion of the transverse processes with the sacrum. While this is mostly asymptomatic, it can be a cause of (referred) pain when pseudoarthrosis develops leading to accelerated degenerative disease. In these cases, increased focal uptake in the pseudoarthrosis on bone SPECT/CT can assist in making the diagnosis (Fig. 7b) (Fogelman et al. 2013).

#### Red flags

Even though guidelines clearly state that routine imaging for low back pain is not considered appropriate, an important exception is made for patients who also report “red flags” symptoms that may conceal malignancy, infection, or other systemic disease (Clearinghouse National Guideline, n.d.). Presence of one of the following symptoms should raise the suspicion of malignancy: a history of cancer, unexplained weight loss, age over 50 years or under 17 years old, failure to improve with therapy, pain persisting for more than 4–6 weeks, night pain or pain at rest. In case of focal hip uptake, SPECT/CT can be particularly useful to differentiate avascular necrosis (Fig. 7c) and osteoarthritis (Fig. 7d) from malignant bone disease (Fig. 7e). Similarly, persistent fever, history of intravenous drug abuse, severe pain, lumbar spine surgery within 1 year, recent bacterial infection, bacteraemia, or an immunocompromised state should prompt a work-up to rule-out occult infection. Both FDG-PET/CT and bone scintigraphy, as well as MRI or CT can be used to detect malignancy. For detecting occult infection, in spondylitis and spondylodiscitis, FDG-PET/CT can be used, especially when suspecting distant spreading (van der Bruggen et al. 2010). In spondylitis and spondylodiscitis the accuracy of using white blood cells (WBC) labeled with either Indium or Technetium is inadequate, with limited penetration of leukocytes and high physiological bone marrow activity, leading to low sensitivity in spinal pathology. In contrast, suspected infection after metallic implants outside of the spine can be visualized with optimal accuracy using WBC and bone marrow imaging (van der Bruggen et al. 2010).

## Important considerations

While many promising results have emerged over the last few years highlighting the potential of bone SPECT/CT, a number of issues require further elucidation. First, a larger evidence base is required to determine if the technique can be used earlier in the diagnostic work-up of bone and joint conditions. This will also need further technical advances to reduce the radiation exposure even further beyond to what is possible today. Technical progress of SPECT/CT has in the last years been impressive, including, in particular, the option to quantify tracer uptake in absolute units, the introduction of semi-conductor detectors, and the development of multimodal image reconstruction methodology (Ritt et al. 2014). Evidence on the clinical value of these technologies in disorders affecting the skeletal system is still scarce, but they represent exciting options to further improve the diagnostic accuracy of bone SPECT/CT, also in the limping patient. Lastly, the cost and availability of SPECT/CT infrastructure within the broader healthcare context will need to be formally addressed.

## Conclusions

Bone SPECT/CT has shown promise to be a valuable problem-solving tool in patients with persistent limping when careful history taking, clinical examination, and first-line imaging modalities fail to identify the underlying cause.
